# Comprehensive lipidomic analysis of the genus *Cutibacterium*

**DOI:** 10.1128/msphere.00054-24

**Published:** 2024-05-07

**Authors:** Anna Chudzik, Mariusz A. Bromke, Andrzej Gamian, Mariola Paściak

**Affiliations:** 1 Hirszfeld Institute of Immunology and Experimental Therapy, Polish Academy of Sciences, Wroclaw, Poland; 2 Department of Biochemistry and Immunochemistry, Wroclaw Medical University, Wroclaw, Poland; Nanjing University of Chinese Medicine, Nanjing, Jiangsu, China

**Keywords:** lipidomics, lipids, fatty acids, UPLC-MS, cutibacteria, *Propionibacterium*, diagnostic markers

## Abstract

*Cutibacterium* are part of the human skin microbiota and are opportunistic microorganisms that become pathogenic in immunodeficient states. These lipophilic bacteria willingly inhabit areas of the skin where sebaceous glands are abundant; hence, there is a need to thoroughly understand their metabolism. Lipids are no longer considered only structural elements but also serve as signaling molecules and may have antigenic properties. Lipidomics remains a major research challenge, mainly due to the diverse physicochemical properties of lipids. Therefore, this study aimed to perform a large comparative lipidomic analysis of eight representatives of the *Cutibacterium* genus, including four phylotypes of *C. acnes* and two strains of *C. granulosum*, *C. avidum*, and *C. namnetense*. Lipidomic analysis was performed by liquid chromatography‒mass spectrometry (LC-MS) in both positive and negative ion modes, allowing the detection of the widest range of metabolites. Fatty acid analysis by gas chromatography‒mass spectrometry (GC-MS) corroborated the lipidomic data. As a result, 128 lipids were identified, among which it was possible to select marker compounds, some of which were characteristic even of individual *C. acnes* phylotypes. These include phosphatidylcholine PC 30:0, sphingomyelins (SM 33:1, SM 35:1), and phosphatidylglycerol with an alkyl ether substituent PG O-32:0. Moreover, cardiolipins and fatty acid amides were identified in *Cutibacterium* spp. for the first time. This comparative characterization of the cutibacterial lipidome with the search for specific molecular markers reveals its diagnostic potential for clinical microbiology.

**IMPORTANCE:**

*Cutibacterium* (previously *Propionibacterium*) represents an important part of the human skin microbiota, and its role in clinical microbiology is growing due to opportunistic infections. Lipidomics, apart from protein profiling, has the potential to prove to be a useful tool for defining the cellular fingerprint, allowing for precise differentiation of microorganisms. In this work, we presented a comparative analysis of lipids found in eight strains of the genus *Cutibacterium*, including a few *C. acnes* phylotypes. Our results are one of the first large-scale comprehensive studies regarding the bacterial lipidome, which also enabled the selection of *C. acnes* phylotype-specific lipid markers. The increased role of lipids not only as structural components but also as diagnostic markers or potential antigens has led to new lipid markers that can be used as diagnostic tools for clinical microbiology. We believe that the findings in our paper will appeal to a wide range of researchers.

## INTRODUCTION

*Cutibacterium* (formerly *Propionibacterium*) is a commonly occurring microbiota that is widely distributed on human skin, and its characteristic ability is the production of propionic acid. These Gram-positive, anaerobic bacteria are opportunistic microorganisms that become pathogenic in states of immunodeficiency (1, 2). Numerous reports indicate the important role of *C. acnes* in the pathogenesis of not only frequent skin diseases such as acne but also the ability to create biofilms on the surface of implants and catheters (3, 4). There are several substrains of *C. acnes* classified into three major genetic lineages, types I, II, and III; some are found in healthy skin (types II and III), and some are associated with diseases, including acne (type IA1) (5, 6). Other cutibacteria are rarely found in opportunistic infections, and *C. avidum* and *C. granulosum* have been described as the causes of soft tissue and medical device-related infections (7). In addition, *C. namnetense* was isolated from surgical samples of human bone infection (8); *C. avidum* was isolated from chronically infected sinuses, ulcers, and abscesses, often in combination with other organisms, but separate cases have also been published (9, 10).

As lipophilic microorganisms, cutibacteria willingly inhabit areas of the skin where sebaceous glands are abundant. The products of their metabolism are proinflammatory factors; hence, there is a need to thoroughly understand the metabolism and structural lipids of these bacteria.

Bacterial lipids share a common backbone composed most often of glycerol, less often of sphingosine, skeletal modifying compounds (choline, ethanolamine, or sugars), and fatty acids. They are the structural components of bacterial cells, and due to the extremely important role of lipids in maintaining the proper functioning of the cell, lipids ensure the durability of their envelope and adaptation to external conditions; lipids also act as energy stores and participate in signal transduction and cell recognition (11). Their composition is a specific fingerprint for individual strains that enable the detection of differences even between phylotypes of the same species (12). In addition to proteomics and genomics, lipidomics has become a tool that allows for a thorough understanding of microbial metabolism (13). In recent years, it has also been proven that lipids exhibit antigenic properties through their ability to stimulate T lymphocytes. Among antigen-presenting cells, CD1a, CD1b, CD1c, and CD1d are involved in the presentation of lipid antigens, including glycolipids of bacterial origin (14, 15). These processes participate in the immune response against microorganisms, which leads to the release of proinflammatory cytokines (16, 17) and may cause pathological changes in the skin or other tissues colonized by bacteria of the genus *Cutibacterium* (18). Hence, the comparative analysis of bacterial lipidomes (profiles of various cellular lipids) increases the understanding of the cellular metabolism occurring in these bacteria but also provides an important basis for determining their interaction with human cells in the future.

This study aimed to obtain broad, untargeted insight into the bacterial lipidome, both in terms of identification and their composition in individual strains, as well as to identify fatty acids with their quantitative composition.

Currently, the leading method in lipid separation and analysis is liquid chromatography‒mass spectrometry (LC-MS). Due to the large variety of properties resulting from the chemical structure and quantitative differences, the analysis of complete bacterial lipidomes is still challenging. These properties are substantial and range from amphiphilic glycerophospholipids through nonpolar glycerolipids to nonionic ceramides (13). Therefore, the identification of lipid compounds based on the combination of high-performance liquid chromatography with tandem MS, which precisely determines the mass and fragmentation data, is an appropriate approach.

Particularly noteworthy in the lipidomic analysis are fatty acids, hydrophobic components of membranes, and a key determinant differentiating lipid structures in bacteria. Therefore, they are good and reliable targets for comparing and characterizing individual microorganisms (19, 20).

Despite the dynamic development of research focused on bacterial lipidomics, comparative lipidomic analyses that allow the compilation of the properties of individual species within the genus, and even within the phylotypes of the same species, are rare. The following work presents for the first time a comparative analysis of lipids and fatty acids obtained from eight different strains of the genus *Cutibacterium:* four strains belonging to *C. acnes* (phylotypes IA1, IB, II, and III), two strains of *C. granulosum* and one strain each of *C. avidum* and *C. namnetense*.

## MATERIALS AND METHODS

### Bacterial strains

Type strains of *Cutibacterium* spp. were obtained from Polish Collection of Microorganisms (PDM), German Collection of Microorganisms and Cell Cultures (DSM), and National Collection of Type Cultures (NCTC; United Kingdom). The origins of the strains are summarized in Table 1.

**TABLE 1:** *Cutibacterium* strains used in the lipidomic analysis

Strain no	Strain name	Origin	Type
DSM 1897	*Cutibacterium acnes* subsp. *acnes*	Facial acne, UK (21)	IA1
DSM 16379	*Cutibacterium acnes*	Contamination of an anaerobic culture (22)	IB
PCM 2334	*Cutibacterium acnes* subsp. *defendens*	Cutaneous abscess (23)	II
NCTC 13655	*Cutibacterium acnes* subsp. *elongatum*	Normal forehead skin, Japan (21)	III
PCM 2401	*Cutibacterium granulosum*	Human acne (24)	-
PCM 2462	*Cutibacterium granulosum*	Culture contaminant (24)	-
DSM 4901	*Cutibacterium avidum*	Unknown source (22)	-
DSM 29427	*Cutibacterium namnetense*	Surgical samples of human bone infection (22)	-

### Growth conditions

All *C. acnes* strains were cultivated in thioglycollate-soy broth (TS, Thioglycollate medium, Merck-Millipore, Darmstadt, Germany) and trypticasein soy broth (Biomaxima, Lublin, Poland) (1:1, vol/vol) under anaerobic conditions (GasPack systems) at 37°C. After thawing from stock solutions stored at −80°C, individual strains were suspended in 5 mL of TS media. To standardize the number of cells subjected to further extraction procedures, the optical density of all cultured strains was measured at a wavelength of 600 nm (OD_600_) at regular time intervals, which allowed us to construct growth curves. The OD_600_ was measured for each strain until a value of 0.64–1.61 was obtained (Table 2), and the incubation time ranged from 48 h to 72 h. This made it possible to capture all the tested microorganisms in the logarithmic growth phase, which allows for a reliable comparison of their lipidomes. Then, from each bacterial culture, a series of 1 mL samples was drawn: the cells were centrifuged, the supernatant (medium) was discarded, and the samples were frozen at −80°C prior to fatty acid methyl ester (FAME) analysis and lipid extraction.

**TABLE 2:** OD_600_ values of *Cutibacterium* spp. after cultivation in thioglycollate-soy broth at 37°C

Strain	OD_600_ value
*C. acnes* DSM 1897, type IA1	0.805
*C. acnes* DSM 16379, type IB	0.965
*C. acnes* PCM 2334, type II	1.41
*C. acnes* NCTC 13655, type III	1.1
*C. avidum* DSM 4901	1.3
*C. granulosum* PCM 2462	1.13
*C. granulosum* PCM 2401	1.61
*C. namnetense* DSM 29427	0.64

### FAME analysis

Bacterial pellets were freeze-dried and subjected to acidic methanolysis (2 M methanolic HCl solution, 1 h, 80°C) with the addition of 40 µg C23:0 fatty acid as an internal standard. Then, 1.5 mL of Milli-Q water and 1.5 mL of hexane were added to each sample and extracted. The samples were centrifuged (4,000 rpm, 10 min) to separate the phases. The upper hexane phase was collected in a separate tube, and the aqueous phase was re-extracted by adding 1 mL of hexane. The hexane phases were combined and evaporated under a stream of nitrogen. Prior to analysis, the obtained FAMEs were dissolved in 100 µL of hexane. The injection volume was 1 µL. Each sample was injected three times.

Fatty acid analysis was performed using a Focus GC with a Zebron ZB-5HT Phenomenex column (30 m × 0.25 mm × 0.25 µm w/5 m Guardian) combined with an ITQ 700 (Thermo Scientific ITQ Series) mass detector. The carrier gas was helium with a 0.3 mL/min flow rate. The mass range was 50–500 *m*/*z*, with positive polarity. Separation gradients in gas chromatography were set as follows: starting at 150°C for 4 min and then increasing by 12°C/min to reach 270°C. The raw mass spectrometry data were processed using Thermo Xcalibur software.

For the annotation of FAMEs and comparison between analytes derived from different bacterial strains, the apex retention time of each detected peak was transformed into a pseudoretention index by interpolation between the retention times of the first and last analytes, *iso*-C15:0 and C23:0 fatty acids, respectively. Next, the peak areas were normalized to the area of the internal standard and the OD_600_ of the sample. Bar plots present the mean (*n* = 3) values of the normalized data. The heatmaps display the median area (*n* = 3) scaled to the median value of the analyte across the experiment and log2-transformed. The calculations and plotting were performed using Excel and R (packages corto and heatmap.2).

### Lipid extraction

The extraction was performed according to a modified method from Bromke et al. (25). Bacterial pellets were extracted using 800 µL of a cold (−20°C) mixture of methyl-*tert*-butyl-ether:methanol (3:1, vol/vol) with the addition of internal standards [0.1 µg/mL deuterated phosphatidylcholine (PC36:0-D_70_) and 0.1 µg/mL deuterated arachidonic acid (AA-D_5_)]. The samples were then sonicated using a cooled (4°C) ultrasonic bath for 10 min. Then, 400 µL of a mixture of water:methanol (3:1, vol/vol) was added to each of the samples, which led to the formation of two polar and nonpolar liquid phases. The phases were collected separately, dried with a speedvac, and stored at −20°C prior to lipidomic profiling.

### Lipidomic analysis

The analysis of the nonpolar phase lipids was performed using a Waters Acquity UPLC system coupled with an Xevo G2 QToF mass spectrometer. The LC conditions were as follows: column CSH C18 reversed-phase column (2.1 × 150 mm, 1.7 µm, Waters); column temp. 60°C; flow rate 300 µL/min; mobile phase A, acetonitrile/water (60:40, vol/vol) with 10 mM ammonium formate and 0.1% formic acid; mobile phase B, isopropanol/acetonitrile (90:10, vol/vol) with 10 mM ammonium formate and 0.1% formic acid; injection volume, 2.5 µL. The MS conditions were as follows: acquisition mode, MS^E^; ionization mode, ESI positive/negative; capillary voltages, 2 kV (positive) and 1 kV (negative); and source temperature. 120°C. Samples were analyzed in positive and negative ionization modes.

The raw acquired chromatograms were converted to .abf files by the Reifycs Analysis Base File Converter with the default settings for the Waters MS^E^ files. For peak detection, alignment, annotation, and peak area integration, the .abf files were loaded into MS-DIAL software (ver 4.9.; RIKEN Institute). The output file was further compared to the in-house database of lipids for refined manual annotation of peaks. All annotated peaks were normalized to internal standard areas and OD_600_. Bar plots present the mean (*n* = 3) values of the normalized data. One-way ANOVA and Tukey’s HSD test were performed to detect statistically significant differences between strains. The heatmaps display the median area (*n* = 3) scaled to the median value of the analyte across the experiment and log2-transformed. The calculations and plotting were performed using Excel and R (packages pcaMethods, corto, and heatmap.2). Loadings and score tables, as well as ordered data presented in heatmaps, are available as research data (see below).

## RESULTS

### Fatty acid analysis

**Fig 1:**
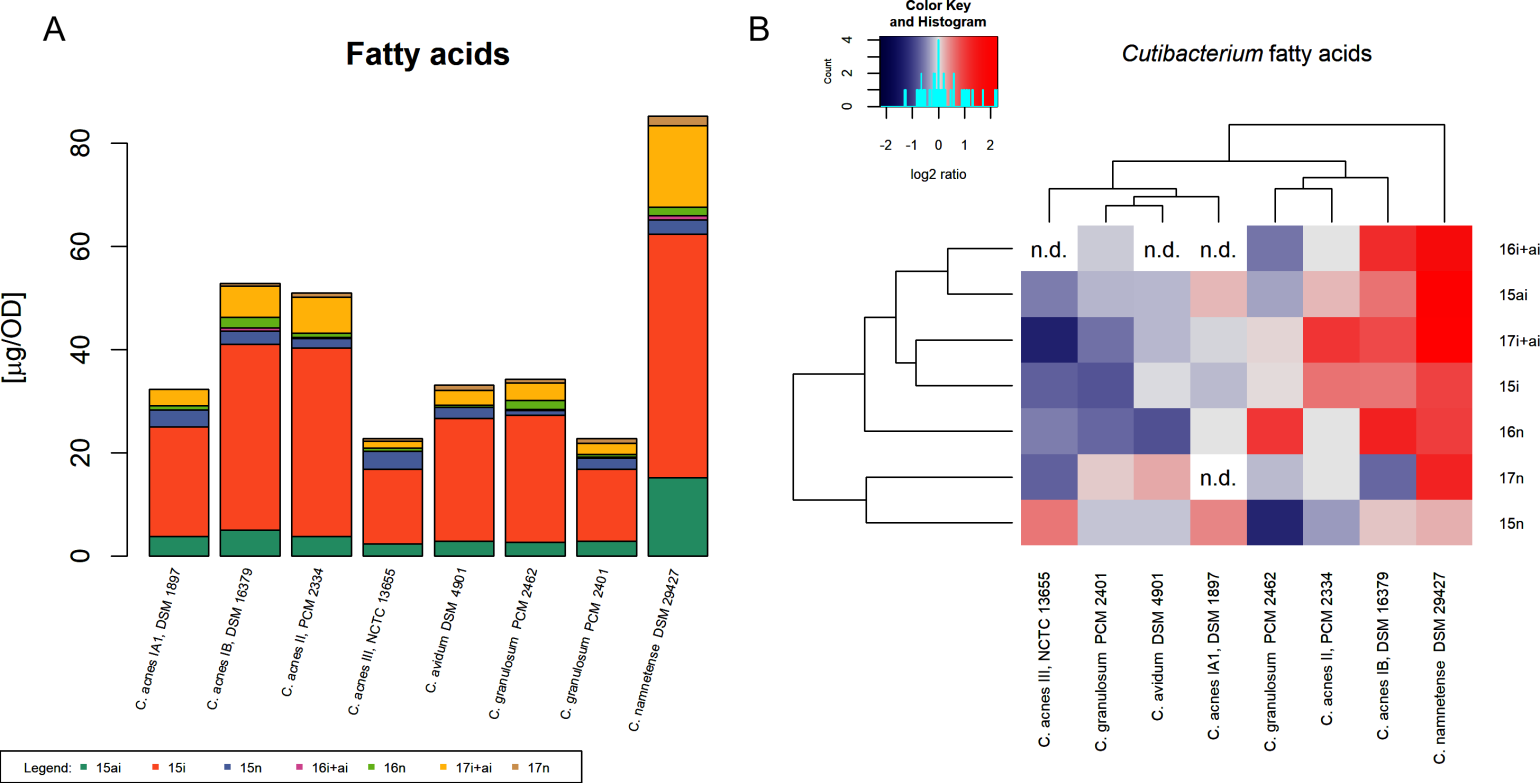
Fatty acid content in *Cutibacterium* spp. (**A**) Bar graph of the quantitative fatty acid content. Individual fatty acids are marked in color: 15i—*iso*-C15:0 methyl-branched, 15ai—*anteiso-*C15:0 methyl-branched, 15n—C15:0 normal, 16i + ai—sum of *iso*-C16:0 and *anteiso*-C16:0 methyl-branched, 16n—C16:0 normal, 17i + ai—sum of *iso*-C17:0 and *anteiso*-C17:0 methyl-branched, 17n—C17:0 normal. *C. acnes* IA1, DSM 1897; *C. acnes* IB, DSM 16379; *C. acnes* II, PCM 2334; *C. acnes* III, NCTC 13655; *C. avidum* DSM 4901; *C. granulosum* PCM 2462; *C. granulosum* PCM 2401; *C. namnetense* DSM 29427. (**B**) Heatmap of fatty acids. The values represent log2 of the ratio to the median of each analyte, and the color key depicts low (blue) to high (red) relative content for each analyte. Cluster analysis is based on Euclidean distances. Analytes that have not been identified are labeled n.d. (not determined).

Comparative analysis of the fatty acid methyl esters of eight representatives of the *Cutibacterium* genus revealed similar fatty acid profiles. They were composed of varying amounts of C15:0, C16:0, and C17:0 saturated fatty acids, among which the most abundant in all strains was *iso*-C15:0 methyl-branched fatty acid (*iso*-C15:0, Fig. 1A). The highest content of fatty acid *iso*-C15:0 was detected in *C. namnetense* DSM 29427 (47.12 µg/OD), and moderate levels were detected in *C. acnes* PCM 2334 (36.04 µg/OD) and DSM 16379 (36.0 µg/OD), while the lowest content was detected in *C. avidum* DSM 4901 (17.23 µg/OD). Cutibacteria was also rich in *anteiso*-C15:0 (ranging from 2.32 to 15.21 µg/OD) and are much less abundant in normal C15:0 (0.97 µg/OD–3.47 µg/OD). The next most abundant fatty acid found was *iso*- and *anteiso*-C17:0. Due to the lack of chromatographic separation of *iso*- and *anteiso*-C17:0 isomers, the content of C17:0 methyl-branched fatty acids is presented as a sum of peak areas (Fig. 1A, 17:0i + ai). *C. namnetense* DSM 29427 contained the highest content of *iso*- and *anteiso*-C17:0 (15.73 µg/OD) fatty acids, whereas *C. acnes* NCTC 13655 had the lowest (1.32 µg/OD). Normal C17:0 acid was present in lower amounts than its branched isomers, ranging from 0.48 µg/OD to 1.94 µg/OD. *Iso*- and *anteiso*-C16:0 fatty acids were, compared to C15:0 and C17:0, the least abundant. Straight chain nC16:0 fatty acid was detected in all analyzed samples, and its contents were comparable to those of nC17:0, ranging from 0.37 µg/OD in *C. granulosum* PCM 2401 to 2.07 µg/OD in *C. acnes* DSM 16379.

Using Euclidean distance-based clustering analysis on log2-transformed ratios, it was possible to distinguish three main clusters of *Cutibacterium* spp. according to their fatty acid profiles (Fig. 1B). The first cluster included two strains of *C. acnes* (DSM 1897 and NCTC 13655), *C. granulosum* PCM 2401 and *C. avidum* DSM 4901. For most of the analyzed fatty acids, these strains contained relatively lower amounts (less than the median of the data set). Interestingly, this group is characterized by nondetectable levels of *iso*- and *anteiso*-C16:0 (with one exception, *C. granulosum* PCM 2401). In addition, nonbranched C16:0 was present at relatively low levels. In the second cluster represented by three strains, *C. acnes* (DSM 16379 and PCM 2334) and *C. granulosum* PCM 2462, the *C. acnes* substrains displayed similarly elevated levels of branched odd-numbered fatty acids (*iso*-C15:0, *anteiso*-C15:0, *iso-* and *anteiso*-C17:0). This is not the case for *C. granulosum* PCM 2462, which also showed relatively low levels of normal C15:0 fatty acid. The *C. namnetense* DSM 29427 species stands out from the remaining clusters because it is characterized by a high content of all fatty acids (Fig. 1B).

### Lipidomic analysis

To obtain as much information as possible on the composition of lipid extracts from eight strains of *Cutibacterium*, mass spectrometry was performed in both positive and negative ionization modes. The mean total ion current was significantly lower in the negative ionization mode, which was reflected by the lower number of annotated lipids. The results obtained from the LC-MS analysis in the positive ion mode allowed for the identification, annotation, and determination of the peak areas of individual analytes for total metabolites. The pool is composed of glycerolipids (52 analytes), glycerophospholipids (8 analytes), sphingolipids (25 analytes), and fatty acid amides (5 analytes).

**Fig 2:**
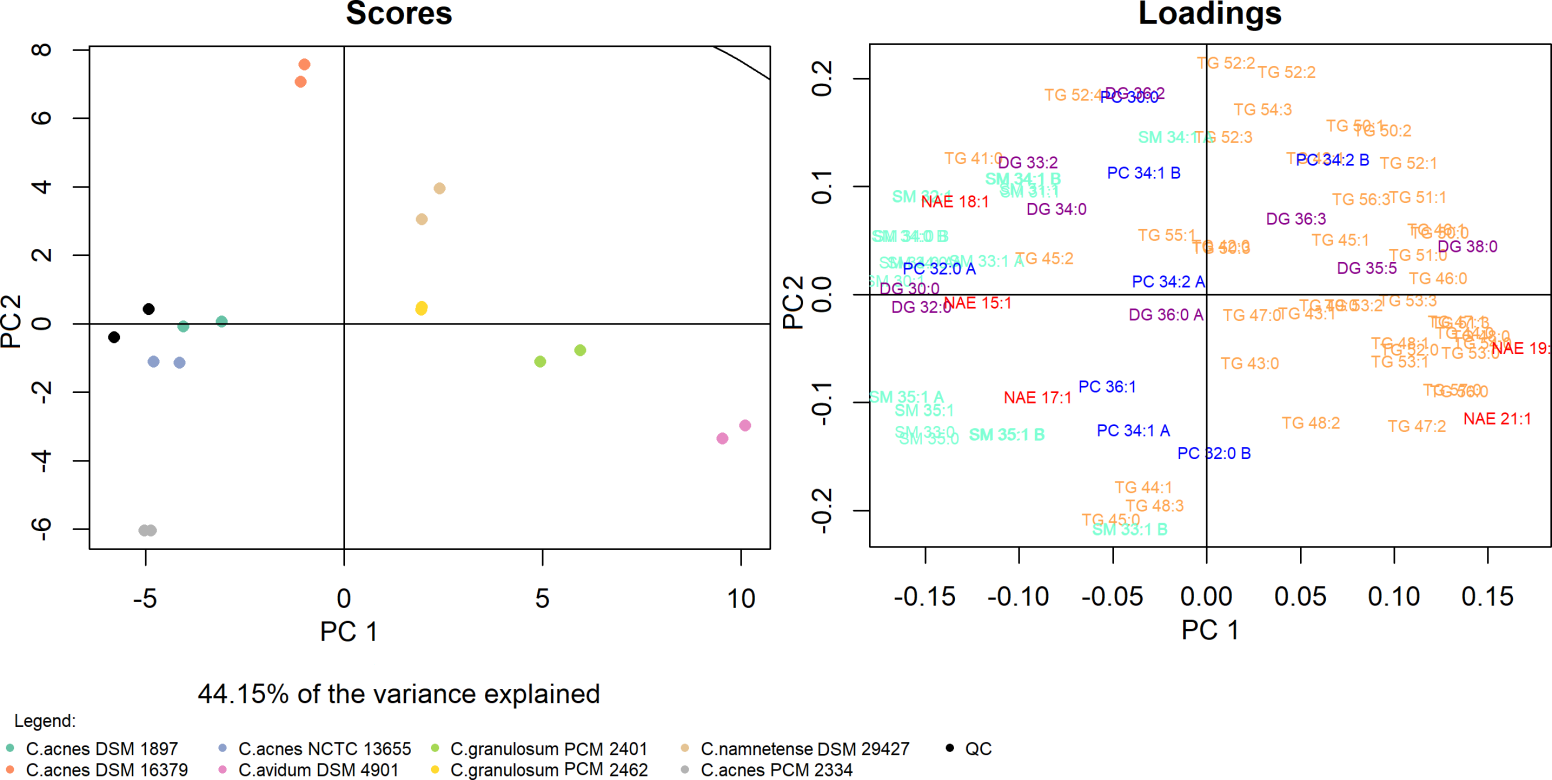
PCA of the lipid profiles of *Cutibacterium* spp. based on 90 annotated lipid analytes in positive ion mode. Each of the two replicates is represented by a colored dot.

Principal component analysis (PCA) is a tool that allows for a thorough comparative analysis of multidimensional data sets such as lipid profiles. The analysis included 90 identified lipid compounds, which highlighted differences and similarities between individual bacterial phylotypes of *C. acnes,* the so-called “acnes group” (Fig. 2). The first principal component and the second principal component accounted for 44.15% of the variance. The analytes that mostly determined this resolution were triacylglycerols (TGs) and sphingomyelins (SMs), especially those located at the extremes of the principal components TG 45:0, TG 46:0, TG 52:2, TG 52:4, TG 54:0 and SM 30:1, SM 33:1 B, and SM 35:1 A (where the letters A and B indicate different chain configurations in the lipid molecule). The “acnes group” is visible through clustering in the first component, especially for the DSM 1897 and NCTC 13655 strains, which are conditioned by similar loadings, such as SM 33:1 A, SM 35:1 A, phosphatidylcholine (PC) 32:0 A, and N-acylethanolamine (NAE) 17:1. The first component also shows the relatively close proximity of the strains belonging to *C. granulosum* PCM 2401 and PCM 2462 while emphasizing the distinctiveness of the only representative of *C. avidum* DSM 4901. The close distribution of the *C. namnetense* DSM 29427 and *C. acnes* DSM 16379 strains is also not surprising—the above data coincide with the clustering shown on the heatmap (Fig. 4).

**Fig 3:**
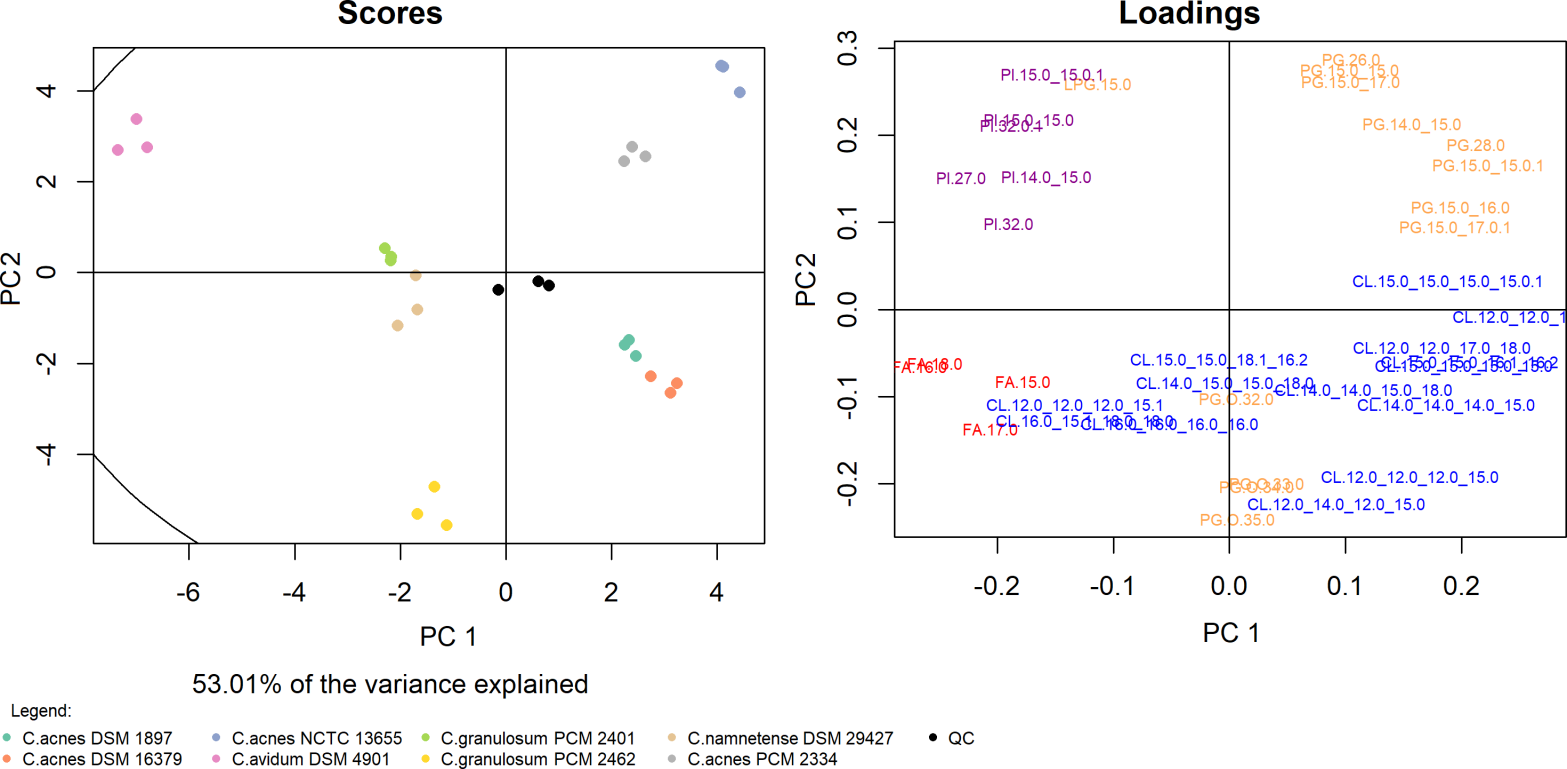
PCA of the lipid profiles of *Cutibacterium* spp. based on 38 annotated lipid analytes in negative ion mode. Each of the three replicates is represented by a colored dot.

The results obtained from the LC-MS analysis in negative ion mode allowed for the identification of 38 analytes (Fig. 3). Due to the lower intensity of the mass spectra obtained in negative ion mode, the analysis was performed in triplicate. The first and second principal components accounted for 53.01% of the variance. The above results indicate the high repeatability of the obtained data while confirming the relationships presented on the heatmap (Fig. 6). Clustering is similar to that shown in PCA in the positive ion mode—the close affinity of the “acnes group” is very clear and is differentiated, especially by the first component. This approach also enabled the observation of interspecies differences, mainly between strains belonging to *C. acnes*, *C. granulosum,* and *C. avidum*. Among the analytes that had a particular impact on this distribution, cardiolipins (CLs) should be distinguished, especially CL 12:0_12:0_12:0_15:0 and CL 12:0_14:0_12:0_15:0. In addition, the fatty acids FA 16:0, FA 18:0, phosphatidylglycerol (PG) 26:0 and PG, which have an alkyl ether substituent (PG O-35:0), contributed significantly.

**Fig 4:**
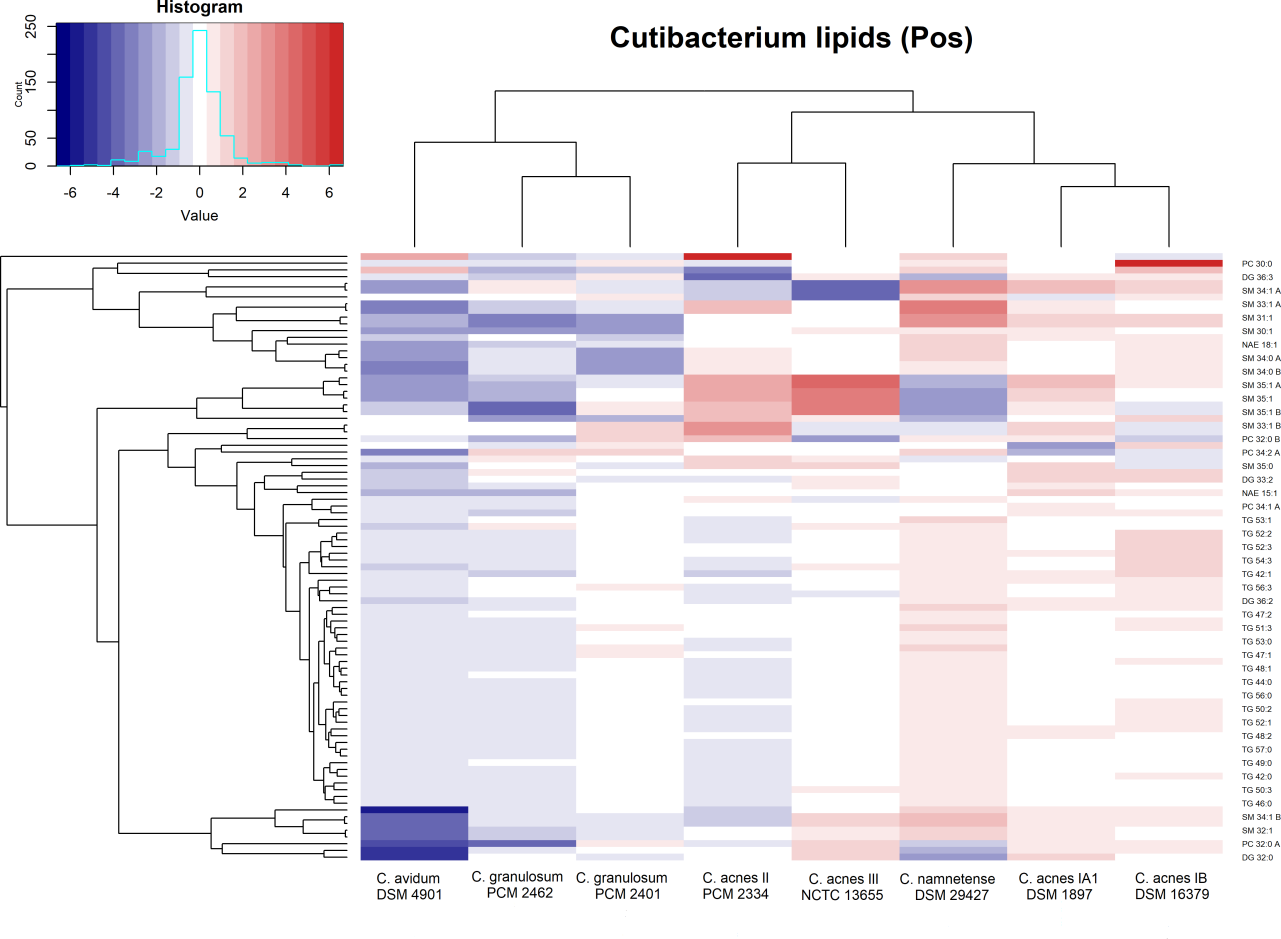
Heatmap of lipid analytes present in *Cutibacterium* spp. measured in positive ion mode. The values represent log2 of the ratio to the median of each analyte, and the color key depicts low (blue) to high (red) relative content for each analyte. Cluster analysis is based on Euclidean distances.

Based on the similarity of the profiles obtained by Euclidean distance clustering in positive ion mode, one can distinguish two main clusters of cutibacterial strains (Fig. 4). The first one contained *C. granulosum* PCM 2401 and 2462 with the more distant *C. avidum* DSM 4901; the second grouped together all four tested phylotypes belonging to the *C. acnes* species with the distinction of close clustering of *C. acnes* PCM 2334 and NCTC 13655 and in the minor cluster *C. acnes* DSM 1897 and 16379 with the more distant *C. namnetense* DSM 29427.

**Fig 5:**
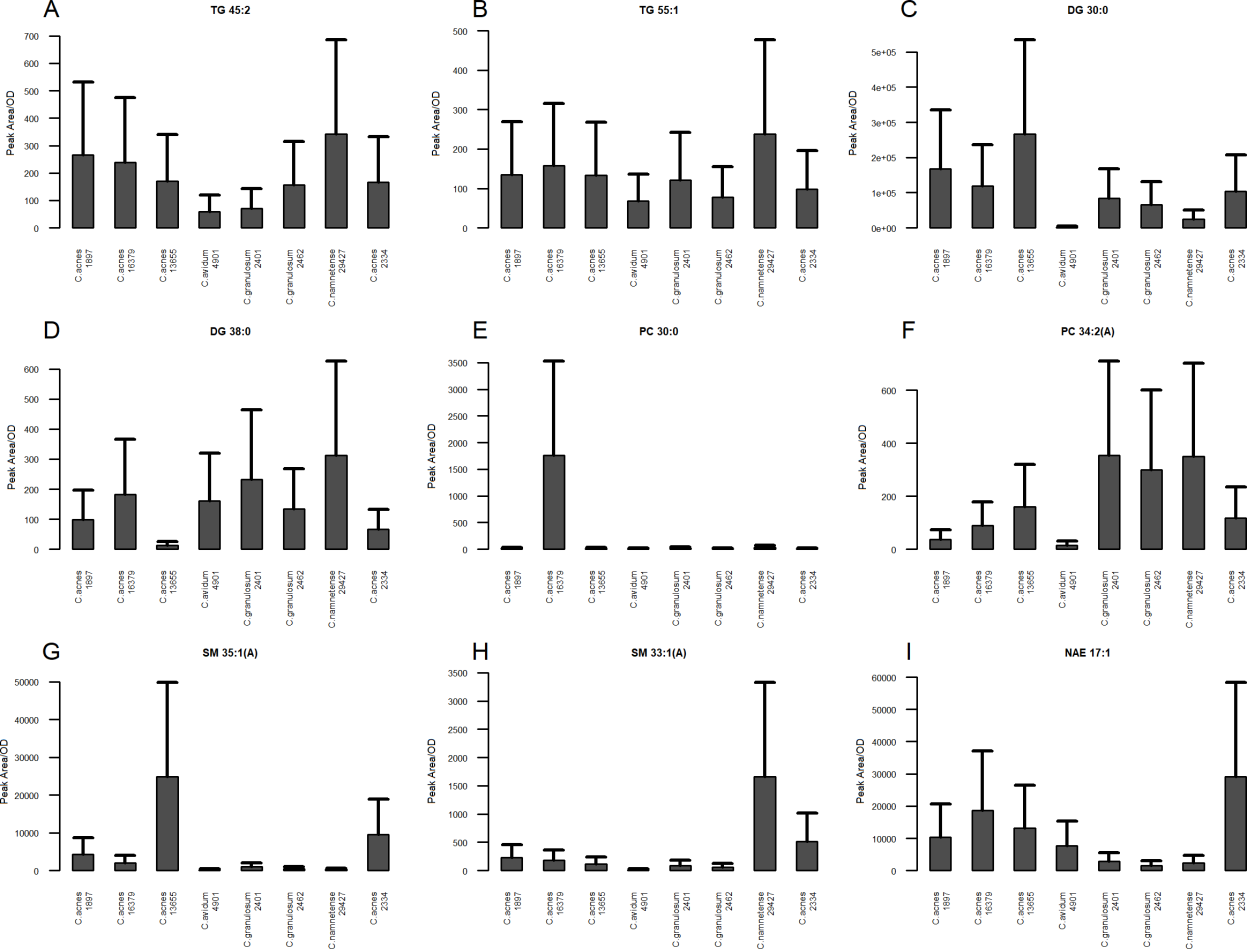
Bar plots of lipid analyte content in *Cutibacterium* spp. measured in positive ion mode: (**A**) TG 45:2, (**B**) TG 55:1, (**C**) DG 30:0, (**D**) DG 38:0, (**E**) PC 30:0, (**F**) PC 34:2A, (**G**) SM 35:1A, (**H**) SM 33:1A, and (**I**) NAE 17:1. The order of *Cutibacterium* in the bar plots was as follows: *C. acnes* IA1, DSM 1897; *C. acnes* IB, DSM 16379; *C. acnes* III, NCTC 13655; *C. avidum* DSM 4901; *C. granulosum* PCM 2401; *C. granulosum* PCM 2462; *C. namnetense* DSM 29427; and *C. acnes* II, PCM 2334.

The glycerolipid class in *Cutibacterium* spp. is represented mainly by triacylglycerols and diacylglycerols (TG and DG, respectively). Among the TGs, 43 analytes were identified with carbon atoms in their acyl chains ranging from 41 to 57 with different desaturation grades—up to four unsaturated bonds. Differences in the quantitative content of lipids belonging to the TG subclass are very clearly reflected by the main clustering visible on the heatmap (Fig. 4), where they appear in the form of two distinct blocks. The characteristic block with a relatively high content of nine TG species (TG 42:1, TG 48:2, TG 50:0, TG 50:1, TG 50:2, TG 51:1, TG 52:1, TG 52:4, and TG 54:3) is located in the middle part of the cluster, which includes the acnes group. These lipids were most abundant in *C. namnetense* DSM 29427 and *C. acnes* DSM 16379 and to a lesser extent in *C. acnes* DSM 1897, which resulted in their clustering. However, in the cluster containing *C. granulosum* PCM 2401 and 2462 and *C. avidum* DSM 4901, there was a clear decrease in the content of TG 44:0, TG 50:2, TG 51:0, TG 54:0, and TG 56:0. The quantitative differences in the above characteristics of the TGs identified in the tested cutibacterial strains are shown in representative bar plots for TG 45:2 (Fig. 5A) and TG 55:1 (Fig. 5B).

In the other subclass of nonpolar glycerolipids, nine DG analytes were identified. It was characterized by the number of carbon atoms in the acyl chain ranging from 30 to 38 and the presence of up to five unsaturated bonds. Particularly noteworthy is DG 38:0, a metabolite found in significant amounts in *C. namnetense* and in lower amounts in other cutibacterial strains, especially *C. acnes* PCM 2334 and NCTC 13655 (Fig. 5D). DG species analysis also provided information on the elevated amount of DG 30:0 in *C. acnes* NCTC 13655 relative to other strains (Fig. 5C). However, it is not the only analyte that distinguishes the “acnes group”—it was also characterized by an elevated DG 32:0 level compared to that of other cutibacteria.

GPs are a heterogeneous class of lipid compounds with phosphatidyl esters attached to the terminal carbon of glycerol. The most abundant PC were PCs, of which we identified eight PCs (PC 30:0, PC 32:0 A, PC 32:0 B, PC 34:1 A, PC 34:1 B, PC 34:2 A, PC 34:2 B, and PC 36:1). In this subclass, a maximum of two unsaturations were observed. Within this entire group of analytes, PC 30:0 (Fig. 5E) stands out because it occurs in significant amounts only in one strain, *C. acnes* DSM 16379. Particularly noteworthy is the metabolite PC 34:2 A (Fig. 5F), which is present in large amounts and is characteristic of both *C. granulosum* strains (PCM 2401 and PCM 2462) and *C. namnetense* DSM 29427.

Among the sphingolipids, 25 compounds belonging to the sphingomyelin (SM) subclass were identified. The length of the SM carbon chains was in the range of 30–35 atoms, and maximum single unsaturation was observed. A particularly spectacular example where SM allows the differentiation of strains from each other is SM 35:1 A (Fig. 5G), the presence of which in significant amounts was detected only in *C. acnes* NCTC 13655. The “acnes group” is also represented by the characteristic SM 30:1 analyte (data not shown), which is almost absent in the other *Cutibacterium* strains tested. Notably, the content of the analyte SM 33:1 A (Fig. 5H) in *C. namnetense* DSM 29427 was significantly greater than that in the other cutibacteria.

The last lipid class of reported analytes in positive ion mode was N-acylethanolamines (NAEs), which are fatty acid amides. Five of these compounds were identified, with acyl chain lengths ranging from 15 to 21 carbon atoms (NAE 15:1, NAE 17:1, NAE 18:1, NAE 19:1, and NAE 21:1). Of particular interest is the NAE 17:1, which distinguishes the “acnes group” together with *C. avidum* DSM 4901 from *C. granulosum* spp. and *C. namnetense* DSM 29427 (Fig. 5I).

**Fig 6:**
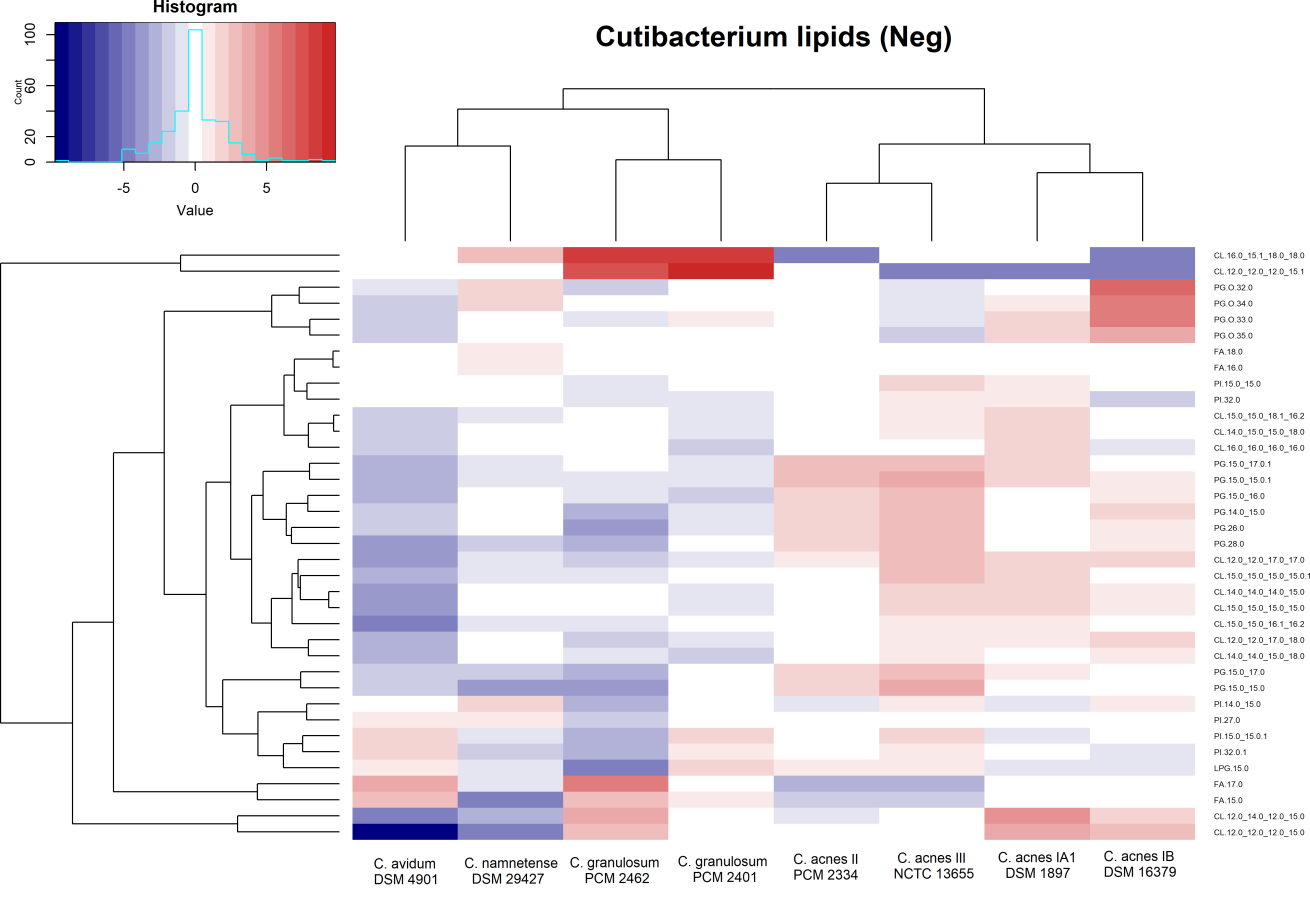
Heatmap of lipid analytes present in *Cutibacterium* spp. measured in negative ion mode. The values represent log2 of the ratio to the median of each analyte, and the color key depicts low (blue) to high (red) relative content for each analyte. Cluster analysis is based on Euclidean distances.

A total of 38 compounds were identified in the negative ion mode. These analytes belong to two classes of lipids: fatty acids (FAs) and glycerophospholipids. The heatmap in negative ion mode (Fig. 6) shows the “acnes group” clustering, which includes the close similarity between the lipid profiles of strains DSM 1897 and 16379 (phylotypes IA1 and IB), as well as between PCM 2334 and NCTC 13655 (phylotypes II and III). The second main cluster also closely resembled both *C. granulosum* strains and *C. avidum* DSM 4901 and *C. namnetense* DSM 29427. The clustering results on the heatmap in negative ion mode were different from those in positive ion mode, where *C. namnetense* DSM 29427 was in the main cluster with the “acnes group.” A particularly visible block is the high content of CL 16:0_15:1_18:0_18:0 and CL 12:0_12:0_12:0_15:1 in both *C. granulosum* strains, which is clearly reduced in the “acnes group.” A high PG content also distinguishes strains belonging to the *C. acnes* subspecies from those belonging to the other tested cutibacteria.

**Fig 7:**
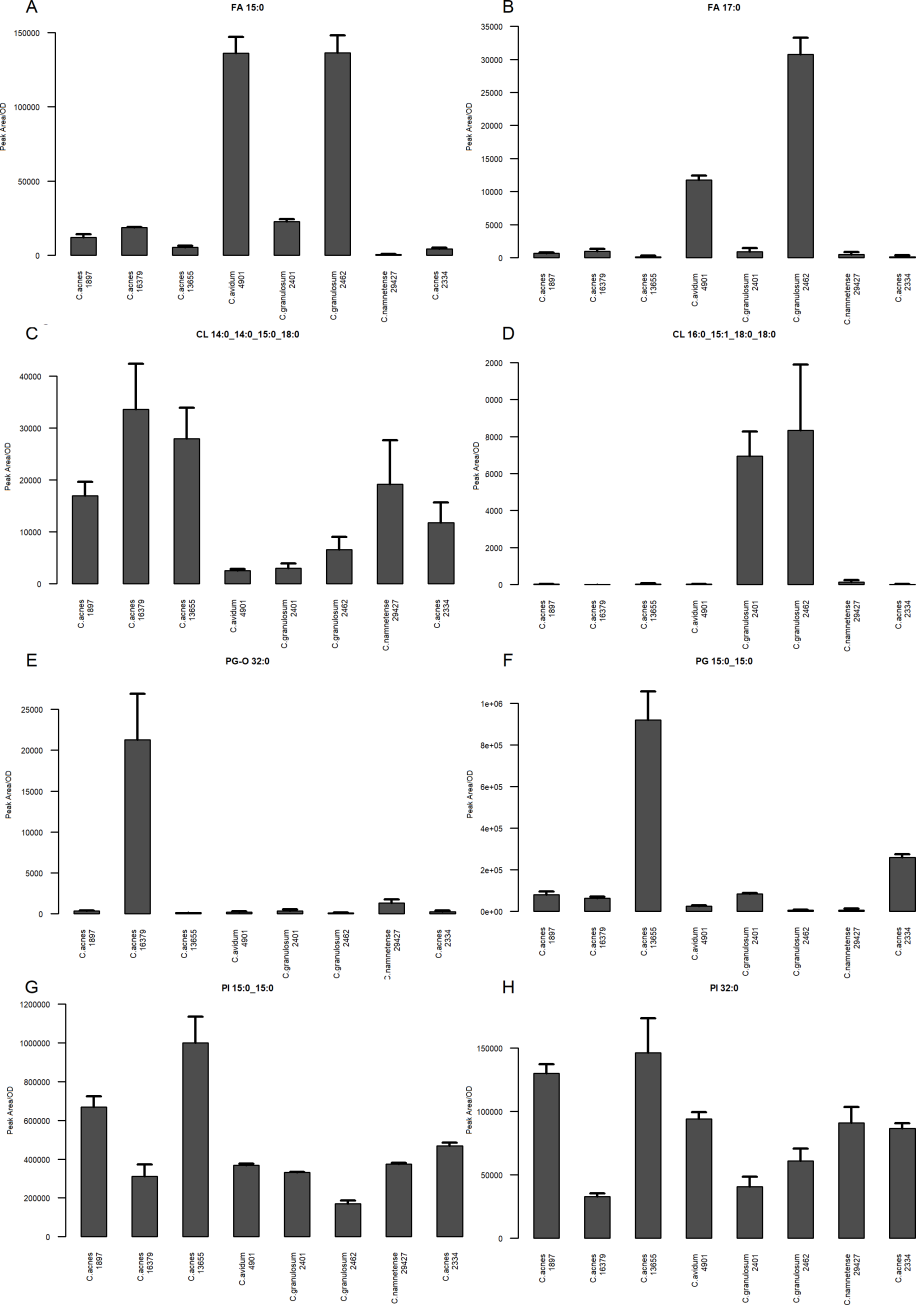
Bar plots of lipid analyte content in *Cutibacterium* spp. measured in negative ion mode: (**A**) FA 15:0, (**B**) FA 17:0, (**C**) CL 14:0_14:0_15:0_18:0, (**D**) CL 16:0_15:1_18:0_18:0, (**E**) PG O-32:0, (**F**) PG 15:0_15:0, (**G**) PI 15:0_15:0, and (**H**) PI 32:0. The order of *Cutibacterium* in the bar plots was as follows: *C. acnes* IA1, DSM 1897; *C. acnes* IB, DSM 16379; *C. acnes* III, NCTC 13655; *C. avidum* DSM 4901; *C. granulosum* PCM 2401; *C. granulosum* PCM 2462; *C. namnetense* DSM 29427; and *C. acnes* II, PCM 2334.

The LC-MS results complement the fatty acid analysis performed by GC-MS, whereas the GC-MS analysis through acid methanolysis produced a picture of all fatty acids bound in various esters. In the LC-MS analysis, we recorded profiles of free fatty acids. The number of carbon atoms in their acyl chains ranged from 15 to 18. Two analytes from this class deserve special attention—C15:0 and C17:0 (Fig. 7B). In both cases, the content was severalfold greater in strains *C. granulosum* PCM 2462 and *C. avidum* DSM 4901. These two factors contributed to the clustering of these two strains, as shown in the heatmap (Fig. 6).

Within the class of glycerophospholipids, cardiolipins (CLs), phosphatidylglycerols (PGs), lysophosphatidylglycerol (LPG), and phosphatidylinositols (PIs) have been identified. In the lipid profile, there were 13 CLs, of which the acyl chains were 13–18 carbon atoms long, and the total number of unsaturated fatty acids among the six fatty acids reached 3. Through analysis of MS/MS spectra, we were able to identify fatty acid-building cardiolipins in *Cutibacterium* spp. Therefore, wherever possible, we used here extended notation of this class, with all four fatty acids separated by an underscore sign (see below). In contrast to the results in the positive ion mode, for CL, no such unambiguous clustering for the “acnes group” was visible. Clear similarities of profiles are observed in this group (e.g., CL 14:0_14:0_15:0_18:0) (Fig. 7C), but this is not as evident as in the case of both *C. granulosum* PCM 2401 and 2462, where metabolites such as CL 12:0_12:0_12:0_15:1 or CL 16:0_15:1_18:0_18:0 (Fig. 6 and 7D) show a very species-specific pattern.

Among the PG subclasses, one lysoPG (LPG), six PGs with an alkyl ether substituent (PG O), and eight regular PGs were identified. In the case of LPG 15:0, this lipid is practically absent from the extract of *C. granulosum* PCM 2462. However, the distribution of PG-O subclass analytes seems interesting—these compounds occur in greater amounts only in the *C. acnes* strains; PG O-32:0, PG O-33:0, PG O-34:0, and PG O-35:0 are analytes characteristic of *C. acnes* DSM 16379 (Fig. 7E). PGs were also dominant in all *C. acnes* strains, especially PG 15:0_15:0 (Fig. 7F) and PG 15:0_17:0.

The last lipid subclass identified was the PI. Among the six analytes, the total length of the acyl chains ranged from 27 to 32 carbon atoms. Similarly to CL, where possible, we used the extended notation of this class, with two fatty acids separated by an underscore sign. A clearly distinguishable lipid was PI 15:0_15:0, whose amount was fourfold to fivefold greater in *C. acnes* NCTC 13655 and DSM 1897 than in the others (Fig. 7G). A similar relationship was observed for PI 32:0 (Fig. 7H).

## DISCUSSION

In recent years, lipidomics, in addition to protein profiling, has been proven to be a useful tool for defining cellular fingerprints, which will allow for the precise identification of microorganisms. Consequently, lipids may become novel molecular markers of bacterial cells (26, 27). To date, most scientific reports have focused mostly on the lipidome of a single (13) and, less often, several bacterial strains (28). By combining GC-MS analysis, which produces a picture of all fatty acids bound to various cellular lipid esters, with LC-MS, which can provide clues about which lipids these fatty acids are bound to, we performed a comprehensive, comparative lipidomic analysis of *Cutibacterium*, which has not been conducted thus far.

Fatty acid (FA) characterization of propionic acid bacteria was previously performed in 1969 (29). Moss et al. described the quantitative predominance of methyl-branched 15:0 fatty acids, followed by normal 15:0, 16:0, and 17:0 fatty acids. FAs longer than 18 carbon atoms in the acyl chain, for example, C20:0 to C23:0, have also been reported to occur but less frequently and in lower amounts. This is confirmed by the work of Cummins et al. in which the composition of FAs in *Propionibacterium propionicum* cells was analyzed, and a quantitative dominance of *iso*-methyl-branched C 15:0 was observed (30). These studies indicate that odd-carbon-chain FAs with a predominance of *iso*-methyl-branched C 15:0 are characteristic of *Propionibacteriaceae*. This explains why the construction of lipids with a specific chain length did not significantly exceed 20 carbon atoms and clearly corresponds to the results of our work obtained through GC-MS and LC-MS analyses in negative ion mode.

The first report that provided insight into the complex lipids of *Cutibacterium acnes* was published in 2018 (31). The observations presented by Jeon et al. are consistent with our results—the most abundant lipid compounds in *Cutibacterium* spp. are nonpolar TGs. We also made similar observations for the length of fatty acid chains present in TGs and the number of unsaturated bonds. These findings also held true for the DG subclass. Among glycerophospholipids, four main subclasses of lipids in both ionization modes have been detected and reported. PCs are lipids that commonly build cell membranes in eukaryotes, but they are quite rare in bacteria. It is estimated that they are found in approximately 15% of prokaryotes (32). It is a lipid characteristic of pathogenic microorganisms, as it has been identified in *Brucella, Bartonella, Pseudomonas, Francisella, Borrelia,* and *Legionella*. Hence, it is presumed that the presence of PCs in bacteria is important for interactions with host cells (32). In our study, we detected seven analytes belonging to the PC and recorded differences in their contents, which allowed us to distinguish between *Cutibacterium* species. The presence of PC in *C. acnes* was also reported in previous studies (31). While the presence of PC in *Cutibacterium* can be considered one of the characteristics of this genus, PGs or CLs are common components that build bacterial membranes (33). Studies indicate that CLs play a significant role in the response of bacterial cells to stress by increasing their quantitative participation in membranes at the expense of the amount of PEs (34). Interestingly, there has been no publication describing the presence of CLs in *Cutibacterium* thus far. In this work, it was possible to identify CLs that had a significant impact on the clustering of strains visible on the heatmap, especially CL 16:0_15:1_18:0_18:0 and CL 12:0_12:0_12:0_15:1, which were highly abundant in both strains of *C. granulosum* and to a lesser extent in *C. namnetense*.

The presence of lipids possessing an ether-linked alkyl chain in propionic acid bacteria was confirmed by Paściak et al. (35). These characteristic and rare compounds, being sugar derivatives of glycerol ethers, were identified for the first time in *Propionibacterium propionicum* PCM 2431. In our study, lipids from the PG subclass that also contained an alkyl ether substituent were identified. These compounds occurred in significant amounts primarily in the *C. acnes* DSM 16379 strain.

The pathway of PI synthesis in bacteria such as *Cutibacterium acnes* was described by Morii et al. (36). The occurrence of PIs has already been reported for *Mycobacterium* and *Actinomycetales, which are* phylogenetically related to *Cutibacterium* (37). We detected six analytes from this subclass in the tested strains, and high amounts of PI (15:0_15:0) were detected, especially in the strains *C. acnes* NCTC 13655, *C. granulosum* PCM 2401, and *C. avidum* DSM 4901.

For many years, it was believed that sphingolipids were not very common in bacteria, except a few, mainly anaerobic genera: *Bacteroides, Prevotella, Porphyromonas, Fusobacterium, Sphingomonas, Sphingobacterium, Bdellovibrio, Cystobacter, Mycoplasma, Flectobacillus*, and *Acetobacter* (38). Nevertheless, ceramides and SMs have been previously identified in *Cutibacterium acnes* (31). SM was the only representative sphingolipid class that was identified in our experiments. Importantly, differences in the contents of SM 33:1 A and SM 35:1 A, which are present in some strains but are virtually absent in others, could be potential lipid markers for these bacteria.

Another analyte found in *Cutibacterium* for the first time was NAE. The role of these bioactive lipids in eukaryotes is well understood, as they exhibit anti-inflammatory and neuroprotective properties (39). They are also called endocannabinoids because they actively modulate cannabinoid receptors. At the same time, its precursor is NAPE (N-acylphosphatidylethanolamine), a phospholipid formed as a result of the transfer of an acyl group chain from an acyl donor to the primary amine ethanolamine moiety of PE (40). To date, little is known about NAE and NAPE in bacteria. It is presumed that NAPE acts as a stabilizer of the cell membrane, preventing damage to the cell membrane and supporting cell division, similar to CLs (41, 42).

Numerous papers have shown that *C. acnes* phylotypes differ not only phenotypically and phylogenetically but also in the expression of virulence factors (43). Therefore, despite *C. acnes* strains occupying the same biological niche to which sebaceous gland-rich skin belongs, each of them (types IA1, IB, II, and III) exhibits pathogenic or commensal potential. Type I strains in particular are described as those most abundantly producing lipases, proteinases, and hyaluronidases, and type IA1 has previously been isolated from acne-prone skin; hence, it is considered a particularly virulent and prevalent strain in conditions such as *acne vulgaris* (44). By analyzing the lipidome of each *C. acnes* strain tested in this study, we were able to observe individual lipid compounds that can be considered markers for a given phylotype. A particular example is *C. acnes* DSM 16379 (type IB), which has a significant amount of PC 30:0. In addition to their obvious structural role, these PCs presumably play a role in virulence determination, thus confirming the pathogenic potential of type I strains (45). Type II and III *C. acnes* are considered to represent “healthy skin” microbiota (46). While some bacterial species are capable of producing sphingolipids, they can also acquire them from a mammalian host. The acquired sphingolipids can then be modified by bacterial enzymes to produce new sphingolipids, which help to conceal microorganisms from the host immune system (47). Particularly noteworthy is sphingomyelin SM 35:1, whose distinct amounts are observed only in *C. acnes* strains PCM 2334 and DSM 13655, which are type II and III, respectively. This finding supports the hypothesis that the acquisition and modification of host sphingolipids may lead to commensalism or hostile interactions (tissue damage/disease) (47).

*C. granulosum* inhabits the same biological niche as *C. acnes* on the surface of seborrheic skin prone to acne, which suggests the existence of a competitive mechanism between them (48). The two *C. granulosum* strains tested were distinguished from other cutibacteria by their high cardiolipin content (CL 16:0_15:1_18:0_18:0), which was absent in the other strains. In studies with another Gram-positive microorganism, *Staphylococcus aureus*, cardiolipin was confirmed to confer virulence to the pathogen by modulating kinase activity (49). The genus *Cutibacterium* is distinguished by *C. avidum*, whose ecological niche is located primarily on moist areas of the skin, especially the axillary region. Mostly, it is considered a commensal microorganism; however, it is also an underestimated opportunistic pathogen (50). According to our studies, *C. avidum* has the highest level of PI 32:0, which can be used as a precursor to produce glycosylphosphatidylinositol (GPI). GPI-anchored proteins can serve as adhesins and may play a role in virulence, especially in fungi and protozoa (51). *Cutibacterium namnetense* was originally isolated from bone infections, and subsequent reports of infections caused by this microorganism confirmed not only its propensity for deep infections but also its antibiotic resistance (52). In the *C. namnetense* strain, we also detected high levels of PI subclass lipids (PI 27:0 and PI 14:0_15:0), which, as indicated above, may be precursors that facilitate invasion in host organisms.

To date, Jeon et al. have performed lipidomic analysis of extracellular vesicles (EVs) released by these bacteria in addition to lipid analysis of *C. acnes* type IA1 (31). Our previous study of the lipid content of EVs from different *C. acnes* phylotypes showed that they have distinct lipid patterns (12). Further studies should also focus on EVs released by other representatives of *Cutibacterium* and compare the lipid content and function of both cells and EVs.

Promising results in the field of lipidomic analysis of *Cutibacterium* spp. constitute a good prognostic perspective for the use of analogous research among other lipophilic microorganisms. Because cutibacteria belong to the Actinobacteria phylum, a similar research approach could be appropriate for these microorganisms. In this regard, representatives of the genus *Mycobacterium* are particularly interesting subjects for lipidomic research due to their unique cell envelope. The lipid content of the *Mycobacterium* wall is very high—up to 40% of the dry cell mass (53). Comparative lipidomic analysis has already been performed within this genus and has enabled the differentiation of *Mycobacterium bovis*, which has monoglycosylated phenolic glycolipids (PGL) termed Mycoside B, from *Mycobacterium tuberculosis*, which has multiglycosylated PGL (54). Recently, a similar approach has been applied to *M. smegmatis*, and it was effective in analyzing other mycobacterial lipids (55).

In summary, our lipid profiling studies of *Cutibacterium* strains aimed to obtain comprehensive insight into the incompletely understood metabolism of these microorganisms. Findings such as strong differences in sphingomyelins or reports for the first time in *Cutibacterium* cardiolipins and N-acylethanolamines derived from odd-carbon fatty acids might help in designing new diagnostic tools for clinical microbiology or help in the selection of new antigenic compounds.

### Conclusions

For the first time, such a broad, comparative analysis of the lipidome of various species from the *Cutibacterium* genus has been presented. We demonstrated that lipidomic studies can provide a precise fingerprint for individual microorganisms. A distinct profile of lipid metabolites allows for differentiation between cutibacterial species and phylotypes of *C. acnes*. The thorough analysis of the profiles enabled the identification of lipids, such as CLs or NAE, which have not been previously detected or reported in *Cutibacterium*. This study provides information about new putative lipid markers with high diagnostic potential in clinical microbiology.

## Data Availability

Supporting data are available at https://ppm.umw.edu.pl/info/researchdata/UMW73b2c2eff1794074b919341340d9016a/.
